# Supplementary material for: Targeting the Cell Wall Salvage Pathway: Dual-Enzyme Inhibition of AmgK and MurU as a Strategy Against Antibiotic Resistance
Source: Int J Mol Sci. 2025 Jul 30;26(15):7368. doi: 10.3390/ijms26157368 (PMC12346966; doi:10.3390/ijms26157368)
Supplement: Supplementary file 1 [file ijms-26-07368-s001.zip › ijms-3666762-supplementary.pdf]

**Supplementary Table S1.** Composition of the Compound Library.

| No. | Name of compound | No. | Name of compound |
|---|---|---|---|
| 1 | (−)-Catechin gallate | 117 | Gastrodin |
| 2 | (−)-Epicatechin | 118 | Gelatin powder |
| 3 | (−)-Epigallocatechin gallate | 119 | Geniposide |
| 4 | (−)-Gallocatechin | 120 | Geniposidic acid |
| 5 | (−)-Gallocatechin gallate | 121 | Genistein |
| 6 | (+)-Catechin hydrate | 122 | Genistin |
| 7 | (±)-Catechin | 123 | Ginkgetin |
| 8 | (±)-Taxifolin hydrate | 124 | Glabridin |
| 9 | 1-{[(5-benzyl-1,3,4-oxadiazol-2-yl)thio]acetyl}-4-methylpiperidine | 125 | H-89 Dihydrochloride |
| 10 | 2,2′,4′-Trihydroxychalcone | 126 | Helichrysetin |
| 11 | 2,4,6-Trimethyl-7-oxo-1,3,5- cycloheptatrien-1-yl 4- chlorobenzoate | 127 | Herbacetin |
| 12 | 2,4,6-Trimethyl-7-oxo-1,3,5-cycloheptatrien-1-yl 4-chlorobenzoate | 128 | Hesperidin |
| 13 | 2,4-Dichlorophenoxyacetic acid | 129 | Hexamminecobalt trichloride |
| 14 | 2,4-Dinitrophenylhydrazine | 130 | Hinokiflavone |
| 15 | 2,5-Diphenyloxazole, scintillation grade | 131 | Hispidulin |
| 16 | 2,6-Dichlorophenol indophenol sodium salt | 132 | Homoplantaginin |
| 17 | 2',7'-Dichlorofluorescein sodium | 133 | Ibuprofen |
| 18 | 2-[(5-benzyl-1,3,4-oxadiazol-2-yl)thio]acetamide | 134 | Icaritin |
| 19 | 2-[(5-benzyl-1,3,4-oxadiazol-2-yl)thio]-N-(2-methyl-5-nitrophenyl)acetamide | 135 | Iodoacetic acid |
| 20 | 2-[(5-benzyl-1,3,4-oxadiazol-2-yl)thio]-N-(5-chloro-2-pyridinyl)acetamide | 136 | Isobavachalcone |
| 21 | 2-[(5-benzyl-1,3,4-oxadiazol-2-yl)thio]-N-(5-methyl-1,3,4-thiadiazol-2-yl)acetamide | 137 | Isoxanthohumol |
| 22 | 2-[(5-benzyl-1,3,4-oxadiazol-2-yl)thio]-N-(5-methyl-1,3-thiazol-2-yl)acetamide | 138 | Kaempferide |
| 23 | 2-{[5-(4-methoxybenzyl)-1,3,4-oxadiazol-2-yl]thio}-N-(5-methyl-1,3-thiazol-2-yl)acetamide | 139 | Kaempferol |
| 24 | 2-Pyridinealdoxime methochloride (2-PAM) | 140 | Kaempferol 7-O-β -d-glucopyranoside |
| 25 | 3,3',5,5'-Tetramethylbenzidine | 141 | Lactose |
| 26 | 3,5-Dinitrosalicylic acid, 98+% | 142 | LAPATINIB DITOSYLATE |
| 27 | 4-Vinylpyridine | 143 | L-Arabinose |
| 28 | 5-Nitroindole | 144 | L-Ascorbic acid |
| 29 | 6-bromochromone-3-carbonitrile | 145 | LASMIDITAN HCl |
| 30 | 7-Isopropoxy-3-phenyl-4H-1-benzopyran-4-one(Ipriflavone) | 146 | Leuprorelin acetate |
| 31 | 9-(4'-Dimethylaminophenyl)-2,6,7-trihydroxyfluorone sulfate hydrate | 147 | L-Homocitrulline |
| 32 | 9-Aminoacridine H\hydrochloride | 148 | Liriodendrin |

| | | | |
|---|---|---|---|
| 33 | ABEXINOSTAT | 149 | L-Ornithine hydrochloride |
| 34 | Acacetin | 150 | Luteolin |
| 35 | Acid Red 94 (Rose Bengal) | 151 | Mangiferin |
| 36 | Acriflavine | 152 | Mannitol |
| 37 | Amentoflavone | 153 | Melezitose |
| 38 | Ammonium bromide | 154 | Melibiose |
| 39 | Amylopectin | 155 | Morin (Morin hydrate) |
| 40 | Angelicin | 156 | Myricetin |
| 41 | Antipain dihydrochloride | 157 | Myricitrin |
| 42 | Apigenin | 158 | N-(6-Aminohexyl)-5-chloro-1-naphthalenesulfonamide hydrochloride (W-7 hydrochloride) |
| 43 | Aprotinin | 159 | N,N-Dimethyl-1-naphthylamine |
| 44 | Astilbin from Engelhardtia roxburghiana | 160 | N,N'-methylene-bis-acrylamide |
| 45 | Auraptene | 161 | Naringenin |
| 46 | AZIMILIDE diHcl | 162 | Naringin |
| 47 | Baicalein | 163 | NDSB-195 |
| 48 | Baicalin | 164 | Neohesperidin dihydrochalcone |
| 49 | Bakuchiol | 165 | N-Hydroxy-1,8-naphthalimide |
| 50 | Bavachin | 166 | Niacinamide |
| 51 | Benzoic acid, 99.5% | 167 | Nicotinamide |
| 52 | beta-carotene | 168 | Nile Blue A |
| 53 | beta-cyclodextrin | 169 | n-Propyl gallate |
| 54 | beta-Naphthoflavone | 170 | OLCEGEPANT |
| 55 | Bismuth nitrate | 171 | OPROZOMIB |
| 56 | BITOLTEROL mesylate | 172 | Orientin |
| 57 | Borane dimethylamine complex | 173 | Orotic acid |
| 58 | Bromocresol green | 174 | Oroxin B |
| 59 | Calcium standard | 175 | p-Aminobenzoic acid(PABA) free acid |
| 60 | Cardamonin | 176 | p-Dimethylaminobenzaldehyde |
| 61 | Cecium Chloride | 177 | Pectolinarin |
| 62 | Cellobiose | 178 | Phenylbutazone |
| 63 | Cetyltrimethylammonium bromide, 99+%(TLC) | 179 | Phenylfluorone |
| 64 | Chlorpromazine hydrochloride | 180 | Phosphorus standard solution |
| 65 | Chrysin | 181 | PIBENZIMOL |
| 66 | Cirsiliol | 182 | Pinoresinol diglucoside |
| 67 | Citric acid, trisodium salt dihydrate, 99.0% | 183 | PIPEQUALINE |
| 68 | COBICISTAT | 184 | POLDINE METHYLSULFATE |
| 69 | Congo red | 185 | Polyvinylpyrrolidone |
| 70 | Costunolide | 186 | Poncirin |
| 71 | Cupressuflavone | 187 | PREXASERTIB diHCl |

| | | | |
|---|---|---|---|
| 72 | Curcumin | 188 | Prinomastat |
| 73 | CYNARIN | 189 | Puerarin |
| 74 | d-(+)-Raffinose pentahydrate | 190 | Pyridoxal hydrochloride |
| 75 | Daidzein | 191 | Pyridoxine hydrochloride |
| 76 | D-alpha-Tocopherol | 192 | Quercetin (Quercetin hydrate) |
| 77 | DANUSERTIB | 193 | Quercetin 3-β-d-glucoside |
| 78 | DARAPLADIB | 194 | Quercitrin (Quercitrin hydrate) |
| 79 | d-Biotin | 195 | RACTOPAMINE HCl |
| 80 | Dehydrocostus lactone | 196 | Raltegravir (Raltegravir potassium) |
| 81 | Deoxycholic acid, sodium salt, 99% | 197 | Rhodamine 6G |
| 82 | Dexamethasone, 98% | 198 | Rhoifolin (Apigenin 7-O-neohesperidoside) |
| 83 | Dextrose, anhydrous, 98.0% | 199 | RIMCAZOLE diHCl |
| 84 | d-Fructose 1,6-bisphosphate trisodium | 200 | RUPINTRIVIR |
| 85 | Dienestrol | 201 | Rutin |
| 86 | Diosmetin | 202 | Saccharose |
| 87 | Diosmin | 203 | Safranine O |
| 88 | Diphenylamine | 204 | Sakuranetin |
| 89 | Dipyridyl | 205 | SALMETEROL |
| 90 | DIQUAFOSOL TETRASODIUM | 206 | Saponin |
| 91 | Dotite·o-Phenanthroline | 207 | Silibinin |
| 92 | d-Pantothenic acid | 208 | Silymarin |
| 93 | d-Sorbitol | 209 | Sinensetin |
| 94 | EPIRUBICIN HCl | 210 | Skullcapflavone II |
| 95 | EPPS(N-(2-Hydroxyethyl)piperazine-N′-(3-propanesulfonic acid)) | 211 | Sodium deoxycholate |
| 96 | ERAVACYCLINE diHcl | 212 | Sodium diethyldithiocarbamate |
| 97 | ETAFENONE HCl | 213 | Sodium m-periodate |
| 98 | Ethyl 4-cyano-3-methyl-5-({[(4-methyl-2-pyrimidinyl)thio]acetyl}amino)-2-thiophenecarboxylate | 214 | Sodium orthovanadate |
| 99 | Ethyl 4-cyano-3-methyl-5-({[(5-phenyl-1,3,4-oxadiazol-2-yl)thio]acetyl}amino)-2-thiophenecarboxylate | 215 | Sodium phosphinate monohydrate |
| 100 | Ethyl 4-cyano-3-methyl-5-{[(2-pyrimidinylthio)acetyl]amino}-2-thiophenecarboxylate | 216 | Sodium pyruvate |
| 101 | Ethyl 4-cyano-3-methyl-5-{[(4H-1,2,4-triazol-3-ylthio)acetyl]amino}-2-thiophenecarboxylate | 217 | Sofalcone |
| 102 | Ethyl 4-cyano-5-[({[5-(2-ethoxyphenyl)-1,3,4-oxadiazol-2-yl]thio}acetyl)amino]-3-methyl-2-thiophenecarboxylate | 218 | Starch azure |
| 103 | Ethyl 4-cyano-5-[({[5-(2-furyl)-1,3,4-oxadiazol-2-yl]thio}acetyl)amino]-3-methyl-2-thiophenecarboxylate | 219 | TAFENOQUINE succinate |

| | | | |
|---|---|---|---|
| 104 | Ethyl 4-cyano-5-[({[5-(3,5-dimethoxyphenyl)-1,3,4-oxadiazol-2-yl]thio}acetyl)amino]-3-methyl-2-thiophenecarboxylate | 220 | TELAPREVIR |
| 105 | Ethyl 5-({[(5-benzyl-1,3,4-oxadiazol-2-yl)thio]acetyl}amino)-4-cyano-3-methyl-2-thiophenecarboxylate | 221 | Tetrabromofluorescein |
| 106 | Ethyl 5-[({[5-(4-chlorophenyl)-1,3,4-oxadiazol-2-yl]thio}acetyl)amino]-4-cyano-3-methyl-2-thiophenecarboxylate | 222 | Thiourea |
| 107 | Famotidine | 223 | TOZASERTIB |
| 108 | FCLA Free Acid (Chemiluminescence Reagent) | 224 | Trehalose |
| 109 | Fisetin | 225 | Triethylamine |
| 110 | Flavanone | 226 | Trifluoperazine dihydrochloride |
| 111 | FLORIFENINE | 227 | UDENAFIL |
| 112 | Fluorescein | 228 | VORUCICLIB HCl |
| 113 | Fluorescent brightnener 28 | 229 | Wogonin |
| 114 | Folic acid | 230 | Xylene Cyanol FF |
| 115 | Formamide, minimum 99% GC | 231 | ZARAGOZIC ACID A trisodium |
| 116 | Galangin | 232 | β-Cyclodextrin |
